# The first physical evidence of subglacial volcanism under the West Antarctic Ice Sheet

**DOI:** 10.1038/s41598-017-11515-3

**Published:** 2017-09-13

**Authors:** Nels A. Iverson, Ross Lieb-Lappen, Nelia W. Dunbar, Rachel Obbard, Ellen Kim, Ellyn Golden

**Affiliations:** 1 Department of Earth and Environmental Sciences, New Mexico Tech, Socorro, NM USA; 2 Vermont Technical College, Randolph, VT USA; 3 New Mexico Bureau of Geology and Mineral Resources, New Mexico Tech, Socorro, NM USA; 4 Thayer School of Engineering, Dartmouth College, Hanover, NH USA

## Abstract

The West Antarctic ice sheet (WAIS) is highly vulnerable to collapsing because of increased ocean and surface temperatures. New evidence from ice core tephra shows that subglacial volcanism can breach the surface of the ice sheet and may pose a great threat to WAIS stability. Micro-CT analyses on englacial ice core tephra along with detailed shard morphology characterization and geochemical analysis suggest that two tephra layers were derived from subglacial to emergent volcanism that erupted through the WAIS. These tephra were erupted though the center of the ice sheet, deposited near WAIS Divide and preserved in the WDC06A ice core. The sources of these tephra layers were likely to be nearby subglacial volcanoes, Mt. Resnik, Mt. Thiel, and/or Mt. Casertz. A widespread increase in ice loss from WAIS could trigger positive feedback by decreasing ice mass and increasing decompression melting under the WAIS, increasing volcanism. Both tephra were erupted during the last glacial period and a widespread increase in subglacial volcanism in the future could have a considerable effect on the stability of the WAIS and resulting sea level rise.

## Introduction

The West Antarctic ice sheet (WAIS), a large and unstable marine-based ice sheet, is highly vulnerable to accelerating ice loss caused by erosion of buttressing ice shelves by upwelling warm ocean water^1^ and could also be impacted by subglacial volcanic eruptions^2, 3^. Understanding the link between glacial cycles and volcanism has been a topic of recent research focus^4–9^, but much remains to be learned on this topic. The delicate balance between ice loss of the WAIS and the potential increase in volcanism, due to increased decompression melting because of ice loss^4, 5^ could cause a positive feedback mechanism that could increase sea level significantly in the future^10^. Subglacial volcanism could also produce a large amount of basal ice melt that would help lubricate the base of WAIS, causing an increase in ice flow, such has already been speculated to have occurred at Pine Island glacier in the past^11^.

**Figure 1:**
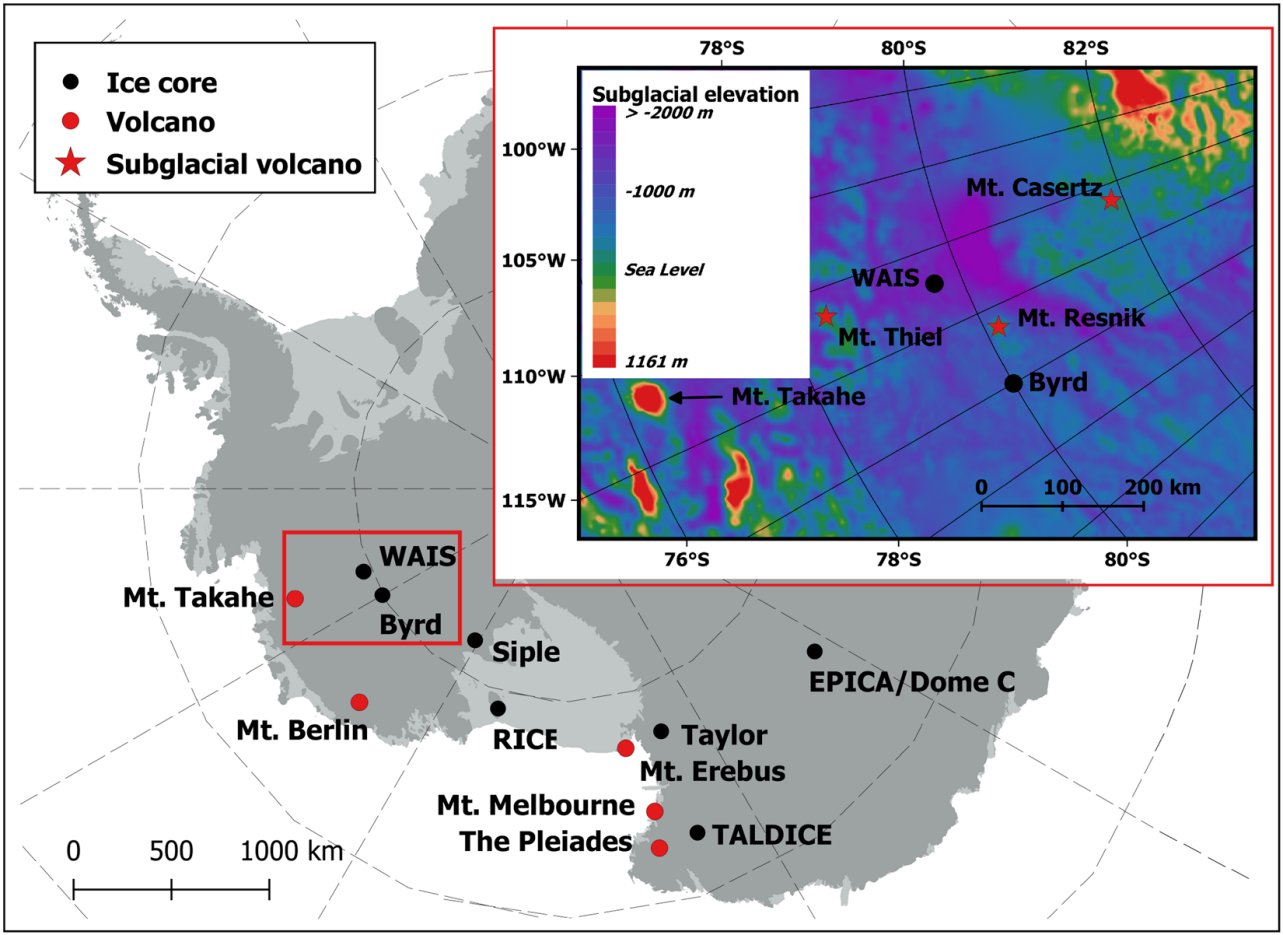
Map of Antarctica with ice cores and active volcanoes. Subglacial bedrock topography map under WAIS with locations of known or inferred subglacial volcanoes (inset). Maps were created using QGIS Version 2.14.3 (http://www.qgis.org/en/site/forusers/download.html) with bedmap2 data (https://www.bas.ac.uk/project/bedmap-2/#data)^46^.

The WAIS is grounded below sea level (in some places by as much as 1200 m) whereas the East Antarctic ice sheet is largely grounded above sea level. The WAIS overlies the volcanically active West Antarctic rift system (WARS) and a number of volcanoes protrude through the ice sheet^12^, or are completely buried beneath the ice^13^ (Fig. 1). Most of the known recent volcanism in West Antarctica (Mt. Takahe and Mt. Berlin) is located near the coast. Other volcanically active areas of the WARS include the McMurdo Sound region (Mt. Erebus) and Northern Victoria Land (Mt. Melbourne and The Pleiades)^12^. Because the eruptive products associated with WARS extension are so inaccessible to study, it is highly likely that unrecognized active volcanism occurs at isolated points in between these larger volcanic provenances and it has been suggested that there are more than 100 subglacial volcanoes under WAIS^14^.

Several studies using remote sensing techniques have suggested the presence of active subglacial volcanism in West Antarctica. A recent volcanic seismic swarm was observed below the Executive Committee Range^2^, young tephra layers have been observed using radar in the Hudson Mountains^11^ and an ice depression at subglacial Mt. Casertz^15^, where seismic activity has also been observed^16^ and has been interpreted to be related to subglacial volcanic activity. However, all of these studies rely on some type of remote sensing, and none were able to directly investigate the physical products of subglacial volcanism. Here, we present the first geochemical and volcanological evidence of subglacial volcanism occurring beneath WAIS obtained directly from associated tephra layers. Furthermore, X-ray micro-computed tomography (μCT) of *in situ* particles in ice-bound tephra layers allows a 3-dimensional view of the distribution and grain size of particles in three distinct and coarse tephra layers from the WAIS Divide ice core (WDC06A). This is the first time volcanic particles in ice have been imaged to see how particle shape and size vary with depth in the ice core, and this may prove to be a useful tool to help understand and characterize the eruptive history and tephra deposition processes. Based on geochemistry, as well as particle size and morphology, two of these layers, which have ice core ages of 22.3 and 44.8 ka (Table 1), are interpreted to be related to eruptions that probably began as subglacial events, and then breached the ice sheet surface, being deposited locally and preserved within the WAIS, then sampled by the WDC06A ice core. The third layer, in contrast, is from a subaerial volcano and shows no signs of water-magma interaction (ice core age of 32.4 ka). The presence of relatively recent subglacial volcanism in West Antarctica highlights the potential threat of West Antarctic volcanism to future ice sheet stability.

**Table 1:** Ice core tephra details

Tephra Layer	Sample Name	Ice core Depth (m)	Layer Thickness (mm)	Tephra Load (g/cm)	Mean Diameter* (μm)	Age (yrs)
Tephra A	WDC06A-2569.205	2569.205	17.5	2.18E-02	85	22,306 ± 290
Tephra B	WDC06A-2871.74	2871.74	11.5	6.62E-03	62	32,397 ± 324
Tephra C	WDC06A-3149.120	3149.12	11.7	7.81E-03	55	44,865 ± 313

Notes: Tephra layers from WAIS Divide ice core. Age is modeled ice core age WD2014^43, 44^. Layer thickness is calculated from lowest tephra within the layer to the highest tephra. Tephra load is calculated from μCT tephra volume measurements (voxels) and the ice core volume. Mean particle diameter is calculated from μCT analyses. Glass densities (not shown) were calculated from the EMPA analyses using Iacovino^45^ Glass Density Cal v3.0 [Microsoft Excel Spreadsheet] using 50% Fe_2_O_3_ and 50% FeO for each layer. *See Supplementary Data for glass densities and calculations.

## Methods

Three visible tephra layers from the WDC06A ice core, studied here, were identified by ice core handlers during initial processing of the ice core. We applied two very different, but complementary, types of analyses to these samples – scanning electron microscopy and electron microprobe analysis of particles filtered from the tephra layers, and μCT of the tephra layers preserved in ice core samples.

At New Mexico Tech, tephra from the layers was recovered by melting the ice, filtering the meltwater and then mounting the samples for imaging and quantitative analysis using the methods of Iverson, *et al*.^17^. Qualitative and quantitative analysis of tephra shards was performed using a Cameca SX100 electron microprobe. Secondary electron microscope (SEM) images were collected using an accelerating voltage of 15 kV, and a probe current of 0.5 nA. Glass analyses were carried out using a 15 kV accelerating voltage, 10 nA probe current, and defocused beam in order to avoid mobilization of Na during analysis. Details of quantitative analysis protocol can be found in Iverson, *et al*.^17^.

Ice core samples (approximately 3 × 3 × 3 cm each) containing the same tephra layers were shipped at −20 °C to the Ice Research Laboratory at the Thayer School of Engineering at Dartmouth College, where they were stored at −30 °C until μCT analysis. Each sample was quartered (vertical cuts) to allow for triplicate sub-sampling of the ash layers. Once cut, each subsample was scanned using a Bruker Skyscan 1172 desktop μCT housed in a −10 °C cold room. The Skyscan 1172 X-ray source produces a fixed conical, polychromatic beam with a <5 μm spot size, an accelerating voltage of 60 kV and a current of 167 μA. Samples were rotated 180° in 0.7° steps, while the X-ray attenuation images were captured by an 11Mp, 12-bit cooled CCD camera fiber-optically coupled to a scintillator.

Reconstruction of the resulting radiographs was completed using a Bruker Skyscan’s NRECON^®^ software that uses a modified Feldkamp cone-beam algorithm to produce a vertical stack of gray-scale cross-section images. The resulting images had a spatial resolution of 15 μm, and selected an internal volume of interest measuring 12 mm × 12 mm in the horizontal plane, with vertical heights varying dependent upon the thickness of the ash layer. Image post-processing was performed using a thermal drift correction of the X-ray source, ring artifact reduction, post-alignment correction, beam hardening correction, and a two-pixel Gaussian kernel smoothing to reduce noise.

A histogram shape-based approach was used to set critical thresholds, enabling us to segment the tephra particles from the gray-scale images. Particles smaller than 27 voxels (cube measuring ~45 microns on edge) were considered noise and excluded from further analysis. Individual three-dimensional analysis was performed on the resulting particles. Statistics were measured on both particle size and particle shape (i.e. sphericity, structure model index, and surface area).

## Results

**Figure 2:**
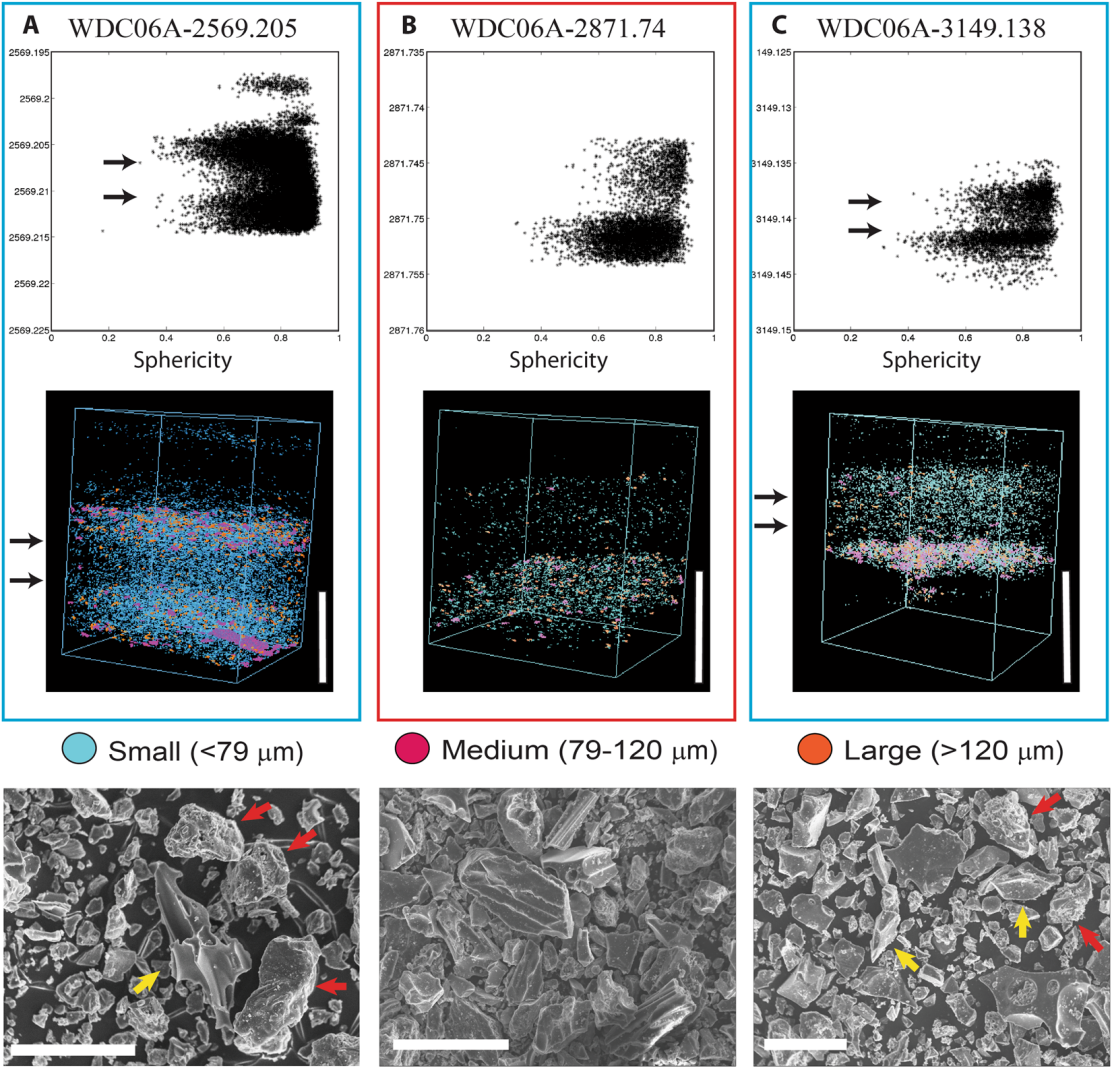
*In situ* micro-CT sphericity and grain size analysis and SEM images of selected tephra layers from WDC06A. Top row- distribution of tephra particle sphericity based on depth in meters (**A**–**C**). Sphericity is a particle shape metric. High sphericity values are more shaped like a sphere (blocky phreatomagmatic particles). Low sphericity values are irregularly shaped particles (more vesiculated and elongated magmatic particles). Black arrows for Tephra A and C represent a change in grain size and sphericity. Middle row- grain size distribution of particles within the ice for Tephra A-C. Vertical white bars equal 1 cm in length. Two layers are considered to be phreatomagmatic (**A** and **C**) with reverse grading and several pulses of larger irregular particles and the other layer (**B**) is magmatic. Bottom row: SEM images of tephra A and C have bimodal particle distribution, large irregular and blocky particles (>100 μm) and small blocky shards (<50 μm). Irregular fluidal particle (yellow arrows) indicative of magmatic eruptions and blocky and mossy (red arrows) particles are typical of phreatomagmatic eruptions. Tephra B contains very large particles (>200 μm) that would be from a large Plinian eruption. White bars are equal to 100 μm.

Visible tephra layers in ice cores typically appear as a faint dark line. In the WDC06A core, three tephra layers, Tephra A, B, and C (Table 1) are uncharacteristically thick (>1 cm). SEM characterization of particles filtered from these layers shows that they are coarse grained (average particle diameter >55 μm). Tephra B contains predominantly magmatic shards, characterized by elongate shapes and presence of vesicles (Fig. 2). Micro-CT imagry shows that large shards appear to be evenly distributed throughout the layer. In contrast, Tephra A and C appear to have a bimodal grain size distribution with most of the particles being <80 μm (blue particles in Fig. 2) and many shards >120 μm (pink and orange particles in Fig. 2). The largest and most irregular particles occur in two distinct bands (bands denoted by black arrows in Fig. 2), one at the base and the other in the upper 1/3 of the layer (Fig. 2). Many of these large fragments have mossy textures (red arrows in Fig. 2) indicative of magma-water interaction^18^, whereas a minority of the large shards are very fluidal, indicative of eruptions driven by magmatic volatiles, and not impacted by external water (yellow arrows in Fig. 2)^19^. These bands are overlain by concentrations of finer, more spherical fragments, corresponding to particles with blocky shapes. Tephra C shows a very similar distribution to Tephra A with large and irregular particles at the base with the second, less well-defined pulse of fine grained blocky particles (black arrows in Fig. 2).

**Figure 3:**
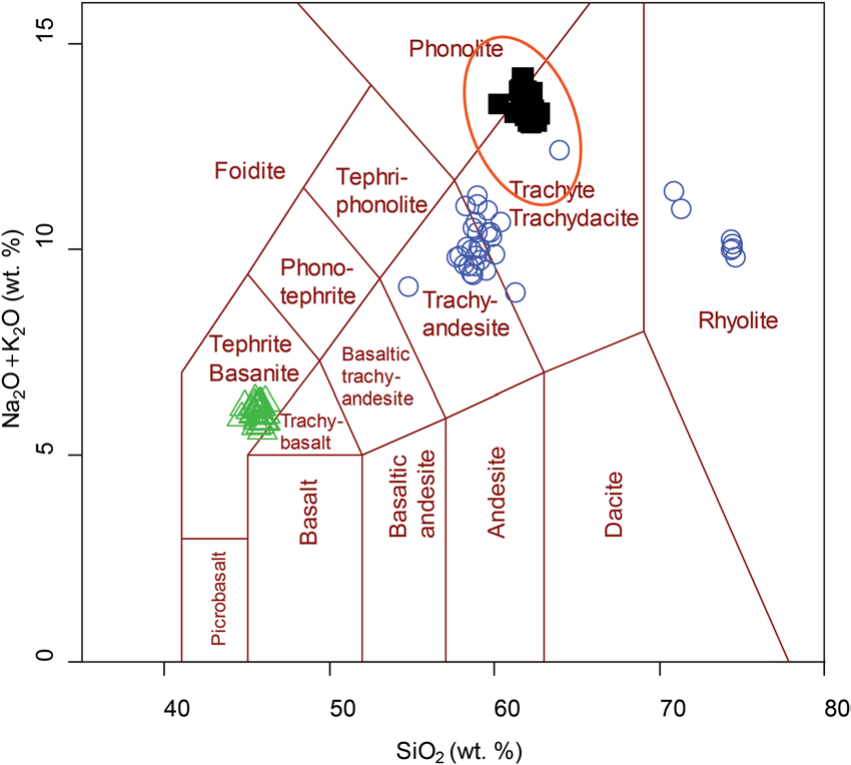
TAS diagram of tephra analyzed by μCT from WDC06A core. Phreatomagmatic tephra layers are blue circles (Tephra A) and green triangles (Tephra C). Black squares represent Tephra B, interpreted to be an ash fall deposit from Mt. Berlin, the dominant tephra producing volcano in West Antarctica. Orange oval represents the typical composition of ice core tephra from subaerial West Antarctic Volcanoes.

Geochemical analyses of individual glass fragments in the tephra, by electron microprobe, show that Tephra A and C are geochemically different from typical ice-bound tephra from Marie Byrd Land volcanoes^20–22^. Tephra A is a compositionally heterogeneous tephra with glass compositions ranging from trachyandesite to trachydacite, with rare rhyolite shards (Fig. 3). Feldspars, which are rare in ice core tephra, are also found within Tephra A, and represent a compositional range (oligoclase to sanidine). Similar geochemical ranges in feldspar have been observed in the Laacher See tephra^23^ and may represent different batches of melt within a given eruption. Tephra C contains homogenous basanitic glass. Tephra B is a typical West Antarctica trachyte that is geochemically consistent with compositions reported for Mt. Berlin pyroclastic deposits^20, 22, 24^.

## Interpretation

Tephra B contains large particles and has been geochemically correlated to the source volcano Mt. Berlin, which is 670 km from the drill site. Based on SEM observation, the morphology of many of the shards in Tephra B are distinctly magmatic, i.e. more vesiculated and less blocky than those observed in Tephra A and C. Furthermore, the composition is trachytic. Mt. Berlin is capable of producing eruption column heights 25–40 km high, which would allow for distribution of large particles over great distances^22^. The 92.5 ka^22^ eruption of Mt. Berlin deposited tephra in marine cores in the Amundsen-Bellingshausen Sea^25^ and East Antarctic ice cores^26^ over 3000 km from the eruptive source. The eruption that produced Tephra B would have been on the same order of magnitude.

The two thick coarse-grained tephra layers (A and C) are interpreted to be from local volcanoes, probably within a few hundred km from the drill site, based on the large, blocky particle size and their unique chemical composition^27, 28^. Neither of these coarse grained, thick tephra are found in the Byrd Core^29^, which is ~100 km away from the WDC06A core site, suggesting a very directional ash cloud, or one that did not travel far from source, allowing substantial deposition at WAIS and none at Byrd. The presence of these tephra layers in the Byrd Core cannot be absolutely ruled out, but, if present, the layer must have been too fine to be recognized by previous workers.

Several lines of evidence point to Tephra A and C being produced by phreatomagmatic eruptions from subglacial volcanoes nearer to the drill site. Tephra A and C have distinct grain size distributions with large mossy and fluidal particles and finer blocky fragments, observed in SEM and in μCT. The fluidal particles are characteristic of magmatic fragmentation^30^, whereas the mossy and blocky particles would be more typical of phreatomagmatic eruption style^30, 31^. In comparison, observations of many other tephra layers found in the WDC06A core are fine grained (<50 μm) and comprised mostly irregular shards 20–30 μm in diameter^17^. Phreatomagmatic eruptions can produce co-erupted magmatic and phreatomagmatic tephra^32^, but if that were the case for these tephra layers, there would be large irregular shards throughout the entire deposit. Instead, there are bands of large irregular shards mixed with smaller blocky fragment followed by band of primarily smaller blocky fragments (denoted by black arrows in Fig. 2). This repetitive sequence suggests mixed phreatomagmatic and magmatic phase followed by a more phreatomagmatic phase and finally a mixed phase. This sequence is likely caused by variations in the amount of water contacting the vent^33, 34^.

Although there are many variables that impact the explosivity of a given volcanic eruption, magmatic composition is a primary controlling factor, with the more evolved and/or H_2_O rich magmas producing higher explosivity eruptions^35^. Therefore, all other things being equal, the less evolved compositions of tephra layers A and C would suggest less explosive subaerial eruptions. The interaction of glacial meltwater with magma during these eruptions would have created episodic explosivity, but would not have contributed to a high, sustained eruption column^36^.

**Figure 4:**
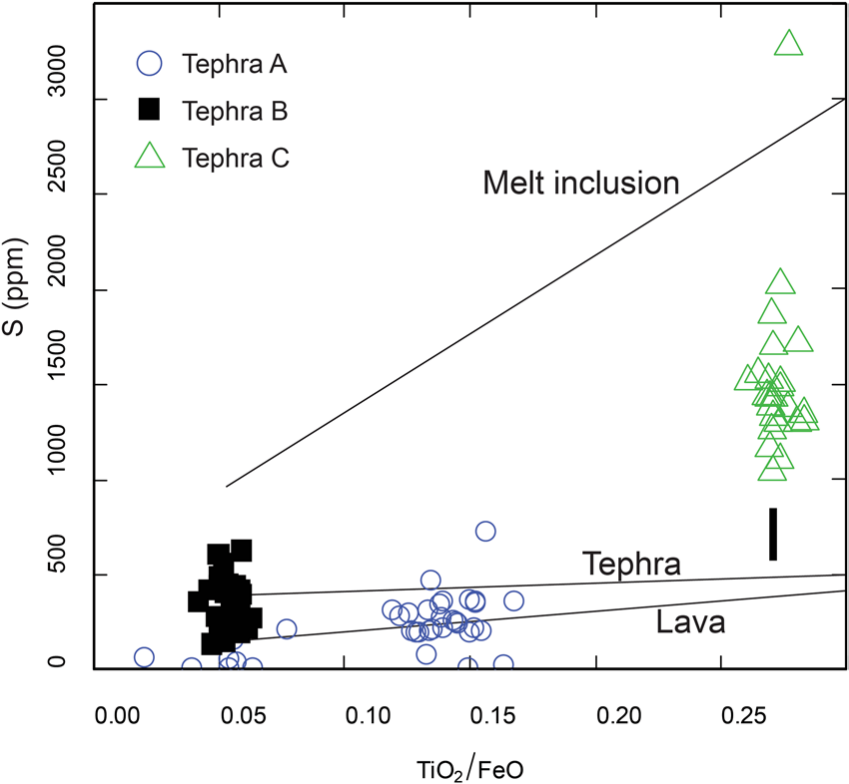
Bivariate plot of S versus TiO_2_/FeO to the assess phreatomagmatic origin of Tephra C. Glass fragments with higher S values are considered to have been erupted prior to degassing as in a phreatomagmatic eruption. Volcanic product best fit regression lines for melt inclusions, tephra, and lava from Icelandic basalts are from Oladottir, *et al*.^38^. Error bar for each S measurement shown in lower right.

A phreatomagmatic genesis of Tephra layer C is further supported by the sulfur (S) concentration in the glass. Sulfur is a volatile component that fractionates into the H_2_O-bearing vapor phase generated during the depressurization that drives volcanic eruptions^37^. During the near-surface, vigorous degassing that takes place during magmatic eruptions, only small amounts of S stay in the melt, so the glass typically contains <20% of the original, pre-eruptive S content^38^. During phreatomagmatic eruptions, S is retained in the glass phase because fragmentation and quenching occur at a depth where S does not fully exsolve from the melt. The basanitic Tephra C, which is compositionally similar to Icelandic basalts, contains elevated and variable concentrations of S (1000–2000 ppm), which is consistent with observations made at Icelandic volcanoes that have undergone both magmatic and phreatomagmatic phases during a single eruption (Fig. 4)^38^. The variable S content of Tephra C, while the other major elements are invariant, suggests that fragmentation occurred at variable depths during the eruption, consistent with the interpretation of a subglacial to subaerial eruption. This tephra may have been generated from an eruption that breached the ice sheet surface, erupted explosively with some mixture of magmatic and phreatomagmatic fragmentation, switched to dominantly phreatomagmatic fragmentation, then had another pulse of mixed fragmentation once the water source was temporarily exhausted. Because Tephra A and B are more evolved and hence have lower S concentrations, S cannot be used to understand changes in fragmentation depth due to lower precision S measurements from the microprobe.

Finding an exact analog to use to understand tephra dispersal for the eruptions that produced the two tephra layers found in the WDC06A ice core that we argue may have a begun as subglacial events, is challenging. However, two well-studied Icelandic analogs are presented here. The first is the 2004 subglacial to emergent eruption of the Grimsvotn volcano. Because the Grimsvotn eruption was basaltic, it is likely to have been somewhat less explosive than the eruptions that produced the two subglacial to emergent WDC06A tephra layers. Albedo changes in the ice cap surface, measured by MODIS, were attributed to tephra deposited on the ice sheet from the Grimsvotn eruption^39^. Based on this information, a tephra load of .01 g/cm^2^, which would represent a fine dusting of tephra, was calculated to be present 30 km from vent. At the other end of the explosivity spectrum, the 1875 rhyolitic eruption of Askja Volcano produced around 2 km^3^ of tephra in a series of eruptions with Plinian, subplinian, and phreatoplinian characteristics^40^. Based on detailed mapping, 5 mm of tephra was present 150 km from the vent, along the maximum dispersal axis for the phreatoplinian part of the event^35^. Given that Tephra A and C in the WDC06A core are likely to be derived from eruptions intermediate in explosivity between these two end members, it is unlikely that the deposition site sampled by the WDC06A core was no more than around 200 km from the eruptive vents. In comparison, the 2010 eruption of Eyjafjallajökull, deposition thickness of >1 cm were found only within 100 km of the edifice^41^^.^

Three subglacial volcanoes near the WDC06A drill site have been identified by aeromagnetic surveys, and are potential sources for Tephra A and C. These are Mt. Thiel, Mt. Resnik and Mt. Casertz^13^. Mt. Thiel is the closest subglacial volcano to WDC06A but is 1.5 km below the ice surface. Mt. Resnik is a tall peak, with the summit only 300 m below the ice surface, <100 km away from WDC06A. However, Mt. Resnik has an overall negative magnetic signature suggesting the edifice is older than 760 ka, the last magnetic reversal^13^. Mt. Casertz is the only subglacial volcano with a current ice depression over the top of the edifice^15^. However, the volcanic peak is 1.4 km below the surface and ~250 km from WDC06A. Because there are two different tephra with distinct chemical compositions it is likely that more than one subglacial volcano has erupted through the ice sheet. All things considered, the best candidate is Mt. Resnik because of the close proximity and low ice burden. Ice sheet elevation models suggest that WAIS was >200 m lower during the last glacial period^42^ reducing the ice burden significantly. It is possible that recent volcanic activity at Mt. Resnik during a time of normal polarity may not be large enough to offset the overall reversed polarity of the edifice. Mt. Resnik may also be the source of both tephra but this is difficult to support without geochemical characterization of material from the Mt. Resnik edifice, which cannot be obtained without drilling.

## Conclusions

Evidence from tephra layers in the WDC06A ice core suggests that two subglacial eruptions have breached the WAIS in the past 45 ka. A basanitic eruption occurred at 44.5 ka and deposited >1 cm of tephra on the local ice sheet. A second, trachyandesite to trachydacite tephra deposited 2 cm of tephra, with some shards greater than 200 μm in diameter, at 22.3 ka. Micro-CT analysis performed on these tephra show a complicated eruption history with a mixed magmatic and phreatomagmatic phase punctuating the predominantly phreatomagmatic eruptions. This is the first time that μCT has been used on *in situ* ice core tephra allowing for the spatial distribution of tephra particles to be observed on a scale not seen before.

Continuing ice loss from WAIS will eventually lower the ice sheet elevation and may cause a positive feedback by increasing volcanism in West Antarctic. Although there is no supporting evidence linking enhanced volcanism with collapse of WAIS in the Quaternary, increased volcanism could produce more basal melt water, increasing ice flow and ice loss thus perpetuating positive feedback. The delicate balance between deglaciation and volcanism may have a profound effect on the stability of WAIS and the subsequent sea level rise.

## Electronic supplementary material

Supplemental Information

Supplementary Data
