# Supplementary material for: The first physical evidence of subglacial volcanism under the West Antarctic Ice Sheet
Source: Sci Rep. 2017 Sep 13;7:11457. doi: 10.1038/s41598-017-11515-3 (PMC5597626; doi:10.1038/s41598-017-11515-3)
Supplement: Supplementary file 1 — Supplemental Information [file 41598_2017_11515_MOESM1_ESM.doc]

**The first physical evidence of subglacial volcanism under the**

**West Antarctic Ice Sheet**

**Nels A. Iverson ^1*^, Ross Lieb-Lappen ^2^, Nelia W. Dunbar ^3^, Rachel Obbard ^4^, Ellen Kim ^4^, Ellyn Golden ^4^**

^1^. Department of Earth and Environmental Sciences, New Mexico Tech, Socorro, NM, USA

^2^. Vermont Technical College, Randolph, VT, USA

^3^. New Mexico Bureau of Geology and Mineral Resources, New Mexico Tech, Socorro, NM, USA

^4^. Thayer School of Engineering, Dartmouth College, Hanover, NH, USA

**Supplementary Information 1.**

*Micro-computed tomography data*

Three samples were analyzed by micro-computed tomography to understand the distribution, shape, and size of particles from *in situ* tephra layers from the WDC06A ice core. Each sample was cut vertically into quarters to allow for triplicate sub-sampling of each ash layer. Once cut, each sample was scanned using a Skyscan 1172 μCT scanner that uses a sealed microfocus x-ray tube with a spot size of 5 μm to produce a fixed conical, polychromatic x-ray source, and is housed in a -10 °C cold room. We set the accelerating voltage of the x-rays to 60 kV with a current of 167 μA. Samples were rotated 180° in 0.7° steps, while the transmitted x-rays were imaged at each step by a 1.3 Mp (1280 x 1024) Hamamatsu CCD camera. We completed reconstruction of the resulting radiographs using Skyscan’s NRECON software that utilizes a modified Feldkamp cone-beam algorithm to produce a vertical stack of gray-scale cross-section images. The resulting images had a spatial resolution of 15 μm, and we selected an internal volume of interest measuring 12 mm x 12 mm in the horizontal plane, with vertical heights varying dependent upon the thickness of the ash layer. As part of image post-processing, we performed a thermal drift correction of the x-ray source, ring artifact reduction, post-alignment correction, beam hardening correction, and a two-pixel Gaussian kernel smoothing to reduce noise.

We used a histogram shape-based approach to set critical thresholds, enabling us to segment the tephra particles from the gray-scale images. Particles smaller than 27 voxels (~0.0001 mm3 or ~ 47 micron cube) were considered noise and excluded from further analysis. We then performed individual three-dimensional analysis on the resulting particles. We measured statistics on both particle size (Fig. S.1-S.3) and particle shape (i.e. sphericity (Fig. S.4-S.6), structure model index, and surface area). Layer thickness was calculated using the average of the triplicate samples. Other metrics (i.e. mean diameter and tephra volume) were used to calculate tephra load and calculations are found in the Supplementary Information excel sheet. The μ-CT data is available upon request.


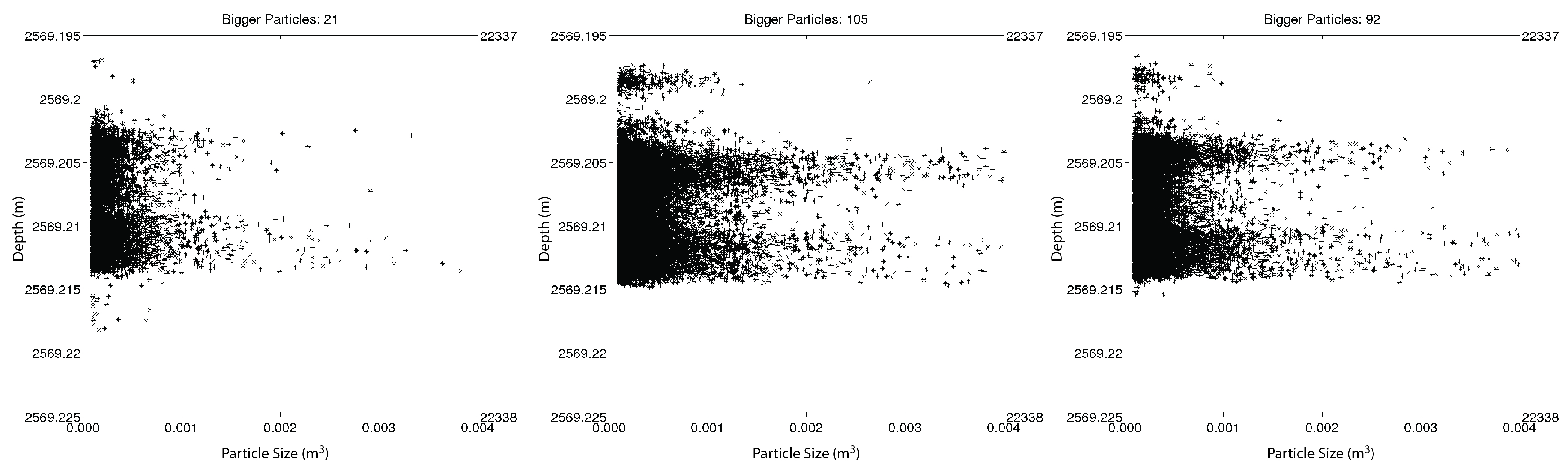


Supplementary Information Figure S.1- Grain size distribution for WDC06A-2569.205. Grain size is in mm^3^. 0.004 mm^3^ is equivalent to a cube that is 158 microns on each side. Bigger particles= particles that are greater than 0.004 mm^3^.


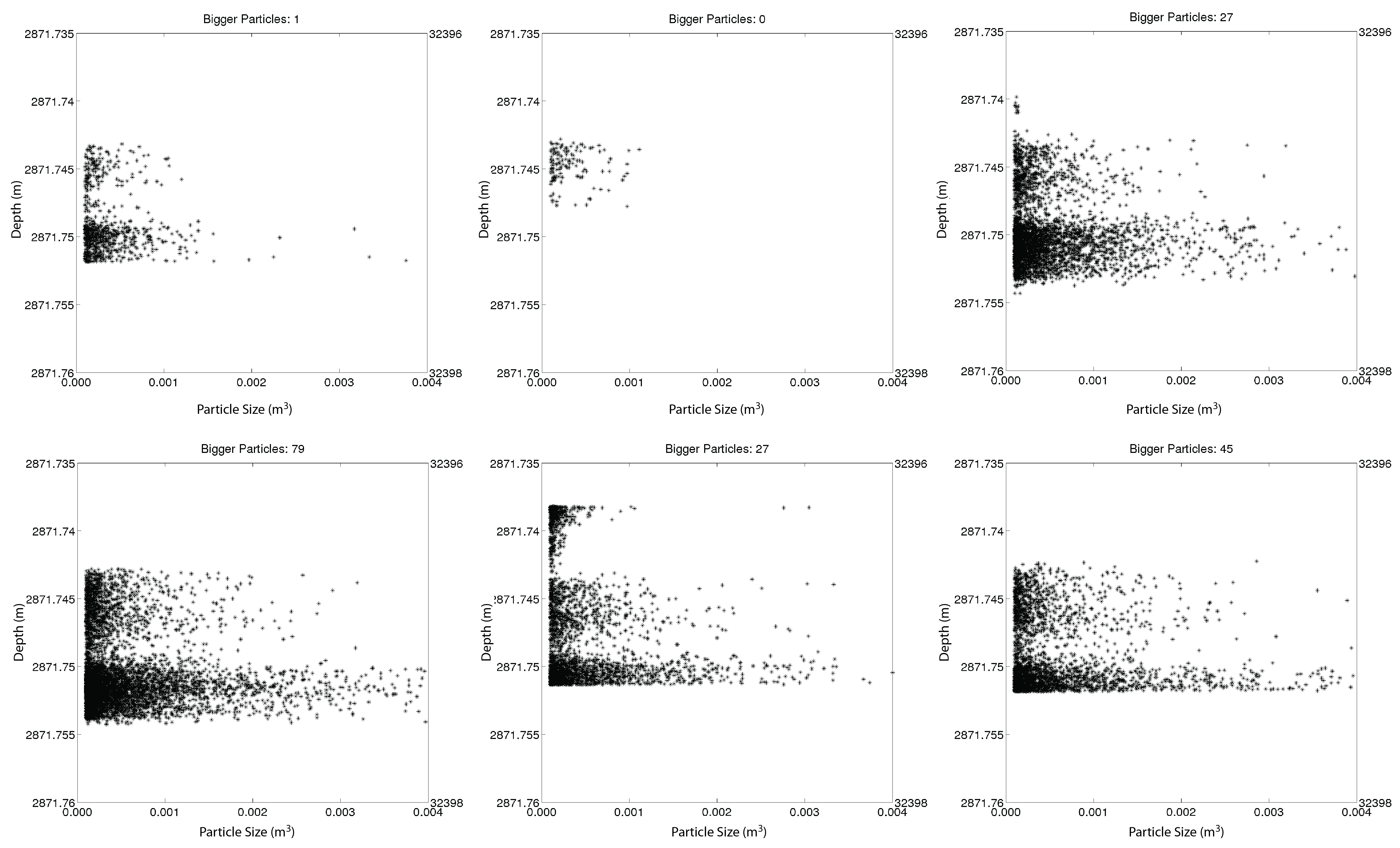


Supplementary Information Figure S.2- Grain size distribution for WDC06A-2871.74. Grain size is in mm^3^. 0.004 mm^3^ is equivalent to a cube that is 158 microns on each side. Bigger particles= particles that are greater than 0.004 mm^3^.


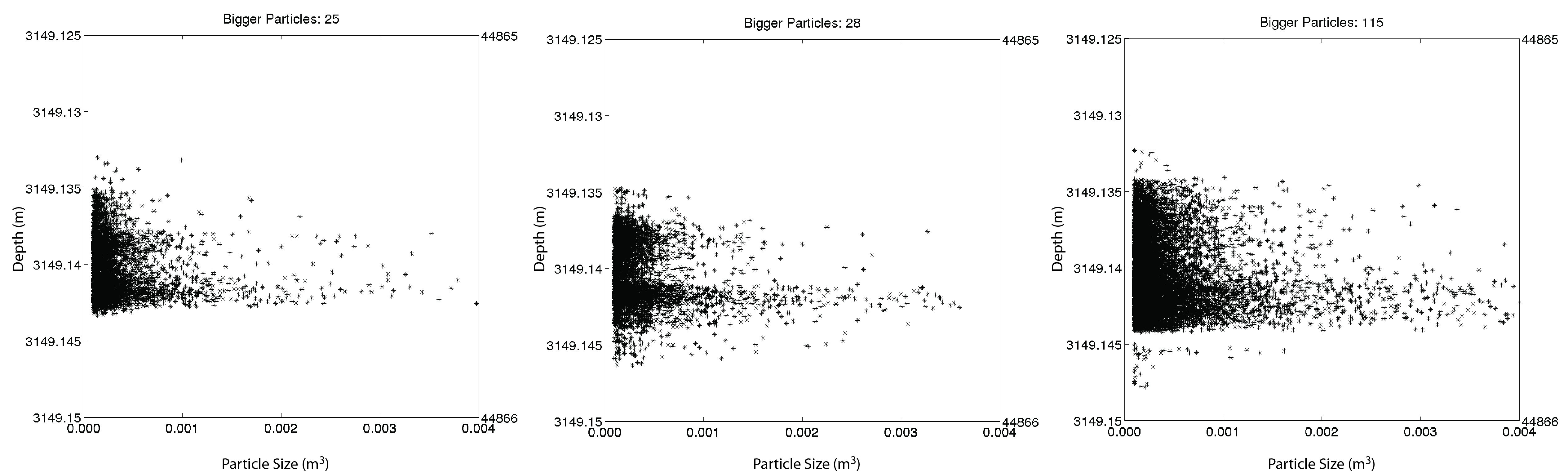


Supplementary Information Figure S.3- Grain size distribution for WDC06A-3149.120. Grain size is in mm^3^. 0.004 mm^3^ is equivalent to a cube that is 158 microns on each side. Bigger particles= particles that are greater than 0.004 mm^3^.


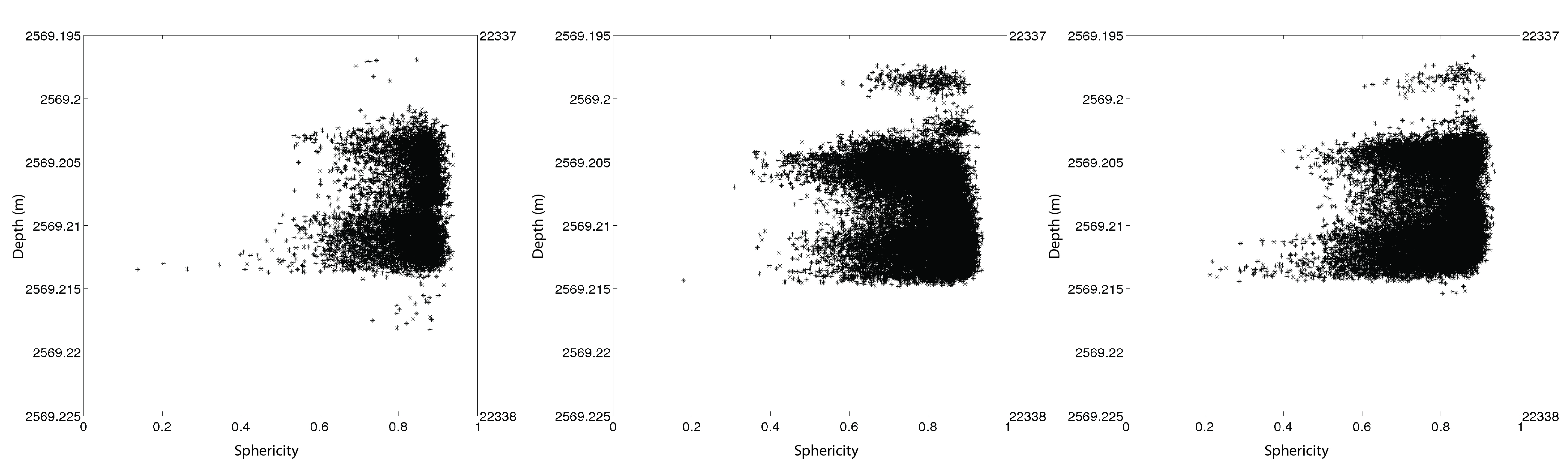


Supplementary Information Figure S.4- Sphericity of tephra particles within WDC06A-2569.205 layer. Sphericity is a metric of how spherical a particle. A sphericity of 1 is a sphere and is similar to blocky phreatomagmatic fragments. Sphericity value <1 are more irregular and the lower the sphericity values the more irregular. Low sphericity values indicate magmatic shards.


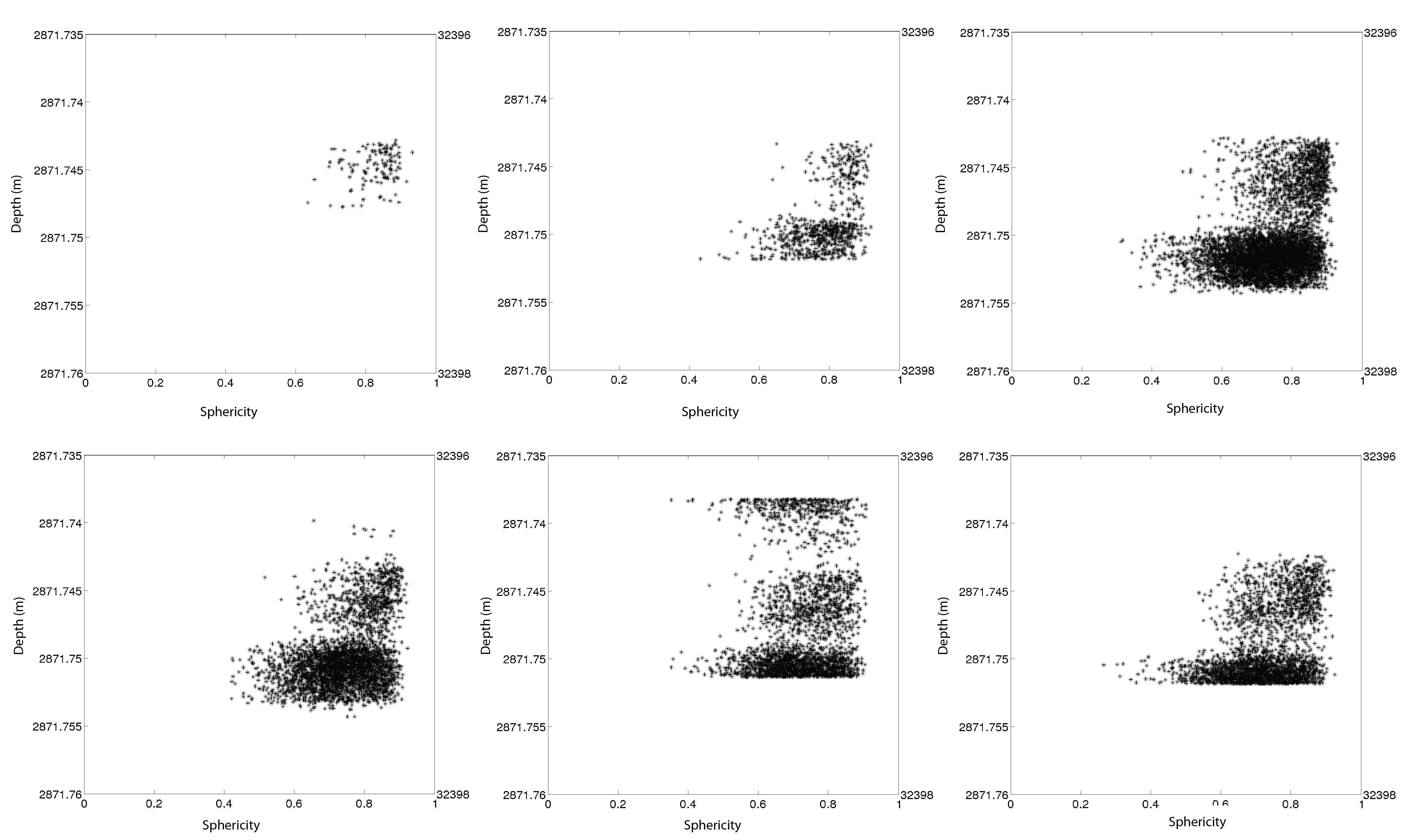


Supplementary Information Figure S.5- Sphericity of tephra particles within WDC06A-2871.74 layer. Sphericity is a metric of how spherical a particle. A sphericity of 1 is a sphere and is similar to blocky phreatomagmatic fragments. Sphericity value <1 are more irregular and the lower the sphericity values the more irregular. Low sphericity values indicate magmatic shards.


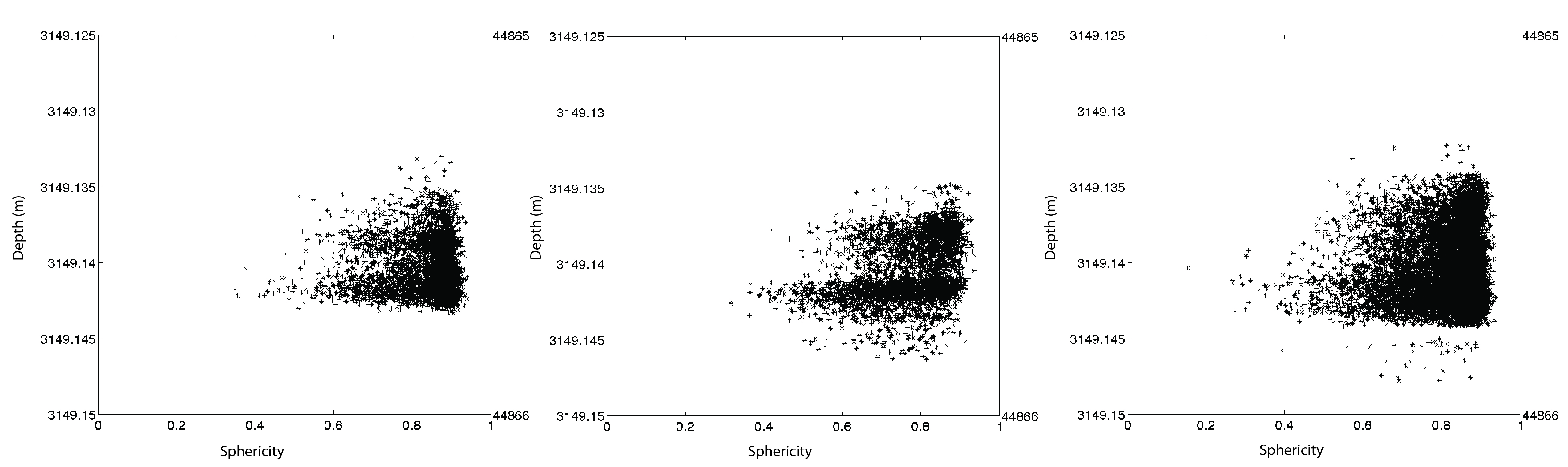


Supplementary Information Figure S.6- Sphericity of tephra particles within WDC06A-3149.120 layer. Sphericity is a metric of how spherical a particle. A sphericity of 1 is a sphere and is similar to blocky phreatomagmatic fragments. Sphericity value <1 are more irregular and the lower the sphericity values the more irregular. Low sphericity values indicate magmatic shards.
